# Discovery of a Novel Sn(II)‐Based Oxide β‐SnMoO_4_ for Daylight‐Driven Photocatalysis

**DOI:** 10.1002/advs.201600246

**Published:** 2016-09-08

**Authors:** Hiroyuki Hayashi, Shota Katayama, Takahiro Komura, Yoyo Hinuma, Tomoyasu Yokoyama, Ko Mibu, Fumiyasu Oba, Isao Tanaka

**Affiliations:** ^1^ Department of Materials Science and EngineeringKyoto UniversitySakyoKyoto606‐8501Japan; ^2^ Center for Materials Research by Information IntegrationNational Institute for Materials ScienceTsukuba305‐0047Japan; ^3^ Graduate School of EngineeringNagoya Institute of TechnologyShowa‐kuNagoyaAichi466‐8555Japan; ^4^ Materials and Structures LaboratoryTokyo Institute of TechnologyYokohama226‐8503Japan; ^5^ Nanostructures Research LaboratoryJapan Fine Ceramics CenterNagoya456‐8587Japan

**Keywords:** divalent tin oxides, first‐principles calculations, high‐throughput screening, low temperature synthesis, photocatalysts

## Abstract

Daylight‐driven photocatalysts have attracted much attention in the context of “green” technology. Although various active materials have been reported and their applications are rapidly increasing, many are discovered after enormous experimental efforts. Herein the discovery of a novel oxide photocatalyst, β‐SnMoO_4_, is demonstrated via a rational search of 3483 known and hypothetical compounds with various compositions and structures over the whole range of SnO‐*M*O*_q_*_/2_ (*M*: Ti, Zr, and Hf (*q* = 4); V, Nb, and Ta (*q* = 5); Cr, Mo, and W (*q* = 6)) pseudobinary systems. Screening using thermodynamic stability, band gap, and band‐edge positions by density functional theory calculations identifies β‐SnMoO_4_ as a potential target. Then a low temperature route is used to successfully synthesize the novel crystal, which is confirmed by X‐ray powder diffraction and Mössbauer spectroscopy. β‐SnMoO_4_ is active for the photocatalytic decomposition of a methylene blue solution under daylight and its activity is comparable to a known photocatalyst, β‐SnWO_4_.

## 1 Introduction

Extensive research has been devoted to exploring new candidate materials for daylight‐driven photocatalysts.^1^, ^2^, ^3^, ^4^ Photocatalysis occurs on the catalytic surface via a combination of complicated reaction pathways, making quantitative predictions of the performance difficult even with leading edge ab initio calculations. However, the photocatalytic activity can be qualitatively understood by the efficiency of forming electron–hole pairs because the creation of electron–hole pairs generally initiates photocatalysis.

The energies of the valence/conduction band edges with respect to photocatalytic reactions (such as water oxidation and reduction) can be used to elucidate the photocatalytic activity. In nontransition metal oxides, the valence band maximum (VBM) is typically composed of the O‐2p states, whereas the conduction band minimum (CBM) is composed of the cation states. The VBM is often modified by choosing *n*s^2^ elements as cations, such as Sn(II) and Bi(III). SnO is one example that shows p‐type conductivity.^5^, ^6^, ^7^

A few oxides of *n*s^2^ elements exhibit high daylight photocatalytic activities, including BiVO_4_,^8^, ^9^, ^10^, ^11^, ^12^ Bi_2_WO_6_,^13^, ^14^, ^15^ and CaBi_2_O_4_ 16 with Bi(III); and Sn_3_O_4_,^17^ SnNb_2_O_6_,^18^, ^19^ Sn_2_Nb_2_O_7_,^18^, ^20^ Sn_2_Ta_2_O_7_,^18^, ^20^ and SnWO_4_ 21, 22 with Sn(II). Among them, β‐SnWO_4_ has been reported to show a superior photocatalytic activity for organic contaminant degradation under simulated daylight compared to BiVO_4_,^22^ which is a promising photocatalyst. The present study aims to explore novel daylight‐driven photocatalysts within Sn(II)‐based complex oxides. The Inorganic Crystal Structure Database (ICSD)^23^ contains ≈60 ternary Sn(II) oxides, but some are duplicate listings and polymorphs. Figure S1 (Supporting Information) shows cation elements with integral valences included in these Sn(II) ternary oxides. These cations can be categorized into three classes: 1A (alkali metal), 4A–6A, and 3B–6B groups. Oxides with alkali metals are unsuited for photocatalysts due to their poor stability in water, whereas oxides with 3B–6B group elements, which are typically comprised of robust oxyanions such as BO_3_^3−^, CO_3_^2−^, SO_4_^2−^, and PO_4_^3−^, tend to show rather wide band gaps. Hence, our research focuses on ternary Sn(II) oxides with *q*A group elements (*q* = 4, 5, and 6) of *n*d^0^ states, SnO‐*M*O*_q_*_/2_, which have a valency of +*q*. The empty d‐band formally contributes to the CBM but not to the VBM.

## 2 Results

### 2.1 Screening via Density Functional Theory (DFT) Calculations

Although the ICSD contains only eight compounds within SnO‐*M*O*_q_*_/2_ pseudobinary systems, we carried out DFT calculations for 3483 SnO‐*M*O*_q_*_/2_ pseudobinary compounds, most of which are hypothetical compounds listed in neither the ICSD nor theoretical databases such as Materials Project Database^24^ and aflowlib,^25^ but their prototype structures and some chemical compositions are registered in the ICSD as ternary compounds. For example, when the AB_2_X_4_‐type structure is listed in the ICSD as a structure prototype, we performed DFT calculations for Sn_2_ *M*O_4_ (*M* = Ti, Zr, and Hf) with the AB_2_X_4_‐type structure. There are 584 for *q* = 4, 201 for *q* = 5, and 362 for *q* = 6 registered prototype structures in the ICSD. In addition, 2, 6, and 6 additional structures were examined for *q* = 4, *q* = 5, and *q* = 6 systems, respectively. Although their prototype structures are not registered, Sn(II) ternary oxides with these structures, such as Sn_2_SO_5_, are registered in the ICSD.

**Figure 1:**
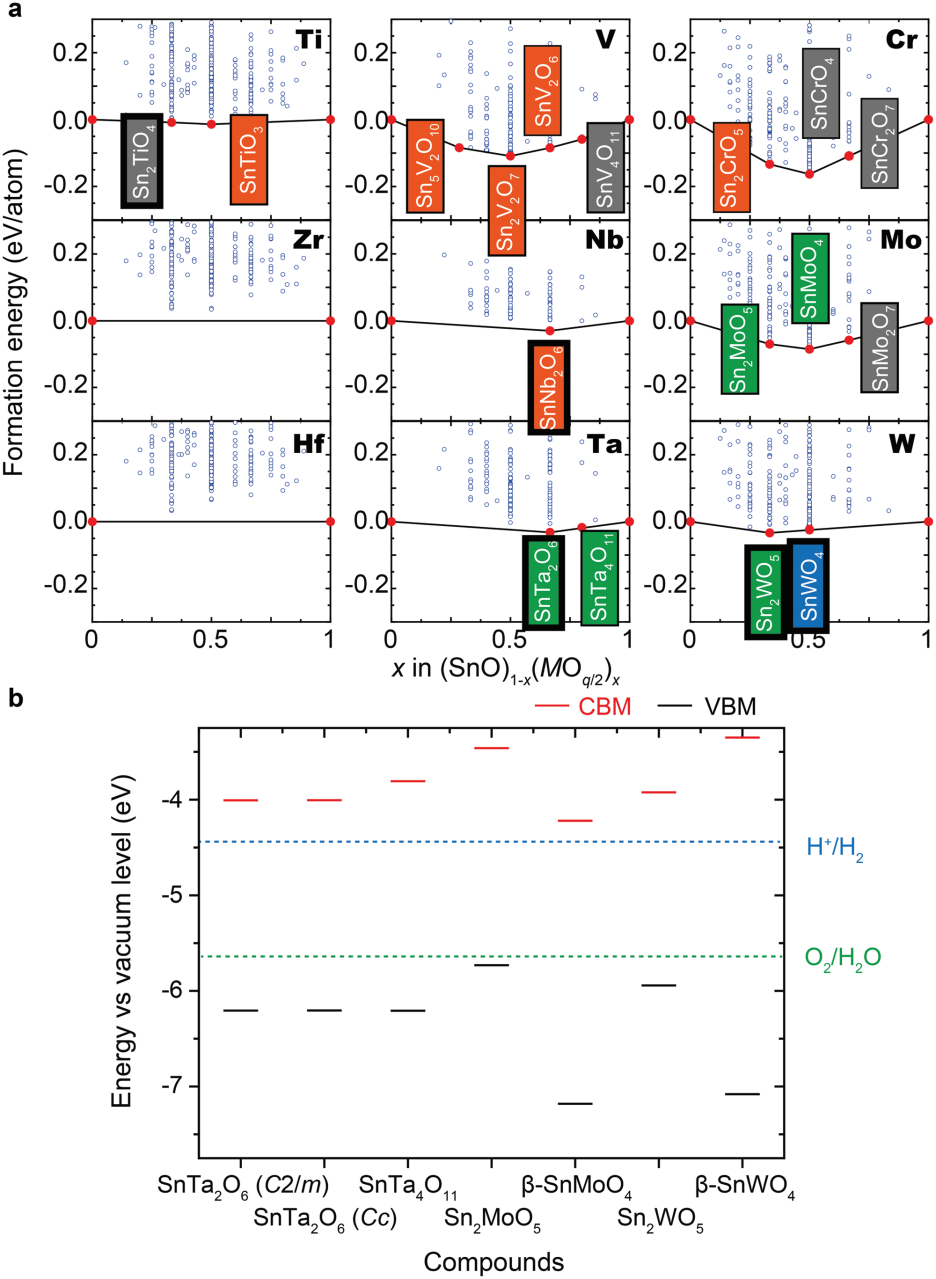
Screening results using DFT calculations. a) Blue circles show formation energy of each compound with many different crystal structures relative to the end‐members (i.e., SnO and *M*O*_q_*_/2_) according to the present DFT calculations. Convex hulls for SnO‐*M*O*_q_*_/2_ pseudobinary systems are shown by black lines. Red circles correspond to compounds located either on or within 5 meV per atom above the convex hull. Thick black frame denote compounds experimentally known and registered in the ICSD. Thin black frame indicates as‐yet‐unknown compounds. Calculated *E*_g_ for the ternary compounds on the convex hull are categorized into four colored regions: gray: 0 ≤ *E*_g_ < 1 eV, orange: 1 ≤ *E*_g_ < 2 eV, green: 2 ≤ *E*_g_ < 3 eV, and blue: 3 eV ≤ *E*_g_. When polymorphs are present, the color corresponds to the largest *E*_g_. b) Calculated VBM and CBM against the vacuum level. Positions of the H^+^/H_2_ and O_2_/H_2_O levels are from ref. 47.

**Figure** **1**a shows the formation energies from the DFT calculations relative to the end‐members (i.e., SnO and *M*O*_q_*_/2_) with the lowest energy structures as well as the convex hull of the formation energy. Among the 3483 ternary compounds, 28 are located either on the convex hull or within 5 meV per atom above the convex hull. Separate calculations using density functional perturbation theory (DFPT) indicate that only one of these compounds has imaginary phonon modes at the Γ point, implying that the other 27 compounds are dynamically stable for the Γ point phonon mode. Hereafter, these 27 compounds are called thermodynamically stable ternary compounds.

Table S1 (Supporting Information) shows detailed information about these compounds. Of the SnO‐*M*O*_q_*_/2_ pseudobinary systems, eight are listed in the ICSD (Table S2, Supporting Information). Of these, six are thermodynamically stable. The exceptions are Sn_2_Ta_2_O_7_, which has the pyrochlore structure, and Sn_3_WO_6_ because they do not meet the present definition of thermodynamically stable compounds. However, these two compounds may be stabilized at a finite temperature (e.g., due to the phonon effect). The remaining 21 thermodynamically stable ternary compounds are not included in the ICSD, indicating that they have yet to be experimentally prepared.

For use as a photocatalyst, the properties related to the electronic structure must be optimized. Because the band gap between the VBM and the CBM, *E*_g_, is a measure of the electronic structure, *E*_g_ can be used to remove compounds unsuited for photocatalysis. In Figure 1a, four colors are used to distinguish the calculated *E*_g_ for the thermodynamically stable ternary compounds (the actual values are listed in Table S1 (Supporting Information)). Seven compounds have *E*_g_ > 2.0 eV, which is the threshold band gap for two reasons. First, the value of *E*_g_ must be greater than the water‐splitting threshold of 1.23 eV plus 0.5 eV to account for the electrochemical overpotentials. Second, experimentally reported Sn(II)‐based photocatalysts such as Sn_2_Ta_2_O_7_ and β‐SnWO_4_ are included in this band gap range.

After screening for the band gaps, additional calculations were performed for selected candidates to evaluate the band‐edge positions relative to the vacuum level. Figure 1b compares the results to the H^+^/H_2_ and O_2_/H_2_O levels, and indicates that all promising candidates are suitable photocatalysts with favorable band‐edge positions, three of which are as‐yet‐unknown [i.e., hexagonal SnTa_4_O_11_ (*P*6_3_22), Sn_2_MoO_5_, and SnMoO_4_]. It should be noted that tetragonal SnTa_4_O_11_ has been reported but its structure is unknown.^26^

Herein our experimental effects focus on the SnO‐MoO_3_ system, especially SnMoO_4_. SnMoO_4_ is isostructural to β‐SnWO_4_, which exhibits a photocatalytic activity for the evolution of H_2_ from an aqueous methanol solution under visible‐light irradiation.^21^, ^22^ Hereafter SnMoO_4_, which is isostructural to β‐SnWO_4_, is referred to as β‐SnMoO_4_.

### 2.2 Crystal and Electronic Structures of β‐SnMoO_4_

**Figure 2:**
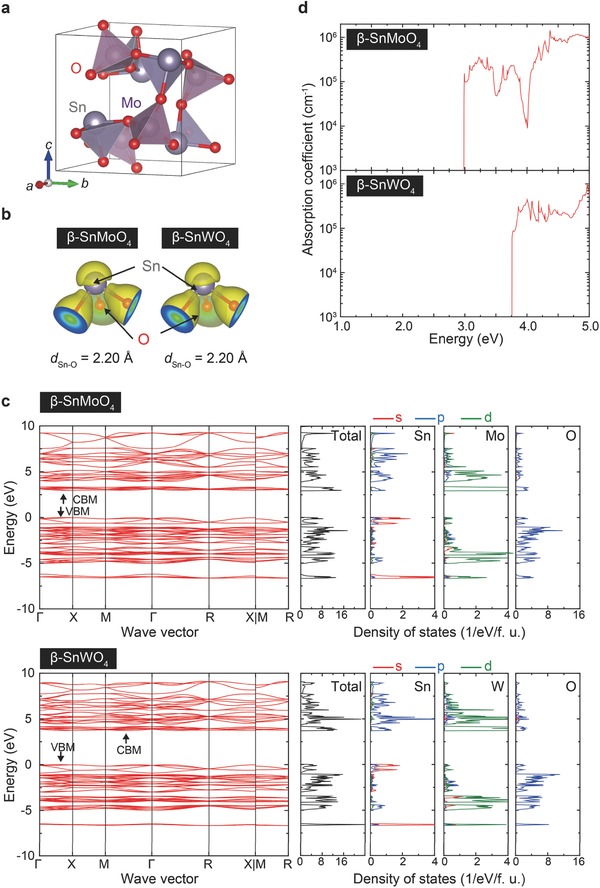
Crystal and electronic structures of β‐SnMoO_4_ and β‐SnWO_4_. a) Theoretical crystal structures of β‐SnMoO_4_ after the structural optimization. b) Charge density isosurface at 0.21 Å^−3^ around the Sn—O trigonal pyramids of both β‐SnMoO_4_ and β‐SnWO_4_. c) Electronic band diagrams along with the total and orbital projected DOS of β‐Sn*M*O_4_ (*M* = Mo and W). VBM is set at 0 eV for each β‐Sn*M*O_4_. d) Calculated optical absorption coefficients of β‐SnMoO_4_ and β‐SnWO_4_.

After screening the compounds by DFT calculations, the crystal and electronic structures of the identified compound β‐SnMoO_4_ were analyzed in depth. β‐SnWO_4_ was also examined for comparison because experimental data are available. **Figure** **2**a shows the theoretical crystal structure of β‐SnMoO_4_ after structural optimization. The calculated lattice constant of cubic β‐SnWO_4_ is 7.584 Å. This value is a 4% overestimate of the experimental value of 7.2989 Å,^27^ which, as discussed below, is similar to the overestimated lattice constant of β‐SnMoO_4_. The calculated lattice constant of β‐SnMoO_4_ (7.543 Å) is close to that of β‐SnWO_4_, which is consistent with the fact that Mo(VI) and W(VI) ions have similar ionic radii (i.e., 55 and 56 pm, respectively, when fourfold coordinated^28^).

β‐SnMoO_4_ is composed of MoO_4_^2−^ tetrahedrons and SnO_3_^4−^ trigonal pyramids that are corner shared. There are four distinct atomic sites: Sn (4a), Mo (4a), O1 (4a), and O2 (12b). Sn atoms are coordinated by three O2 atoms to form a trigonal pyramid. The Sn—O bond length of 2.20 Å is almost the same as that in β‐SnWO_4_. Such an asymmetric coordination environment is common in Sn(II) oxides such as SnNb_2_O_6_ and α‐SnWO_4_, and is a fingerprint for the formation of a lone pair. Mo is located near the center of the tetrahedron. Figure 2b compares the charge density around the Sn—O trigonal pyramid at the charge isosurface of 0.21 Å^−3^ for β‐SnMoO_4_ and β‐SnWO_4_. Both have a nonbonding charge density around the Sn atoms, confirming the presence of a lone pair.

The electronic band structures of β‐SnMoO_4_ and β‐SnWO_4_ are shown in Figure 2c. β‐SnMoO_4_ (β‐SnWO_4_) shows an indirect band gap of 2.96 (3.73 eV). In both compounds, the VBM (CBM) is located at a low symmetry point along the Γ‐X (M‐Γ) lines. The difference between the indirect and direct gaps is only a few meV due to the very small band dispersion. The minimum direct gap is 2.96 eV for β‐SnMoO_4_ and 3.74 eV for β‐SnWO_4_, which are almost identical with their respective indirect gaps. The total and orbital projected density of states (DOS) of β‐SnMoO_4_ and β‐SnWO_4_ in Figure 2c indicate that the VBM in both compounds is composed of Sn‐5sp and O‐2p orbitals. The main component of the CBM is Mo‐4d or W‐5d with a small contribution of Sn‐5sp. The Bader charge analyses consistently indicate that the valency of Sn is 2+.

Figure 2d shows the calculated optical absorption coefficients of β‐SnMoO_4_ and β‐SnWO_4_. Both spectra show a steep increase near the threshold, corresponding to the electronic transition energy over the minimum direct gap. This feature should be useful for efficient light absorption, and hence, advantageous for photocatalysis.

### 2.3 Experimental Synthesis of β‐SnMoO_4_

The synthesis of any crystalline oxide composed of Sn(II) and Mo(VI) has yet to be reported to the best of our knowledge. Although one study claims to have synthesized SnMoO_4_ 29 for a humidity sensor application, the crystal structure was not analyzed in detail, and only a simple X‐ray powder diffraction (XRD) was reported. According to our experiments, a mixture of rutile‐type SnO_2_ and monoclinic MoO_2_ (slightly distorted rutile type structure) should be formed via the synthesis conditions in ref. 29 (i.e., 998 K for 12 h in an inert atmosphere). Indeed, the XRD profile in ref. 29 can be assigned by superposition of these two crystals as shown in Figure S2 (Supporting Information).

**Figure 3:**
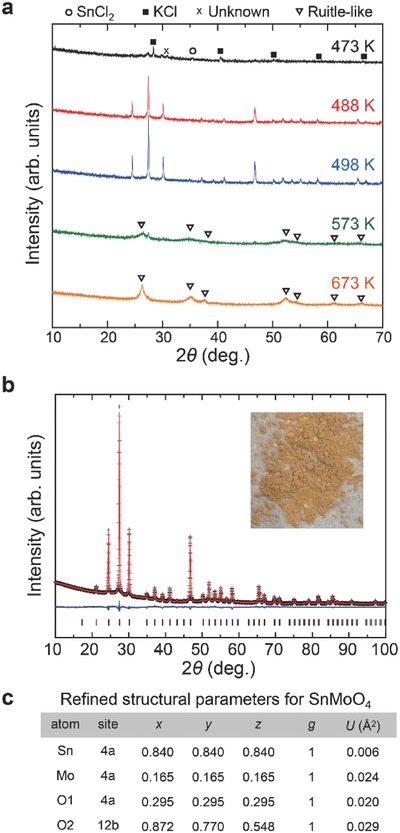
XRD profiles and crystal structure analysis of β‐SnMoO_4_. a) XRD profiles of the samples obtained at a constant temperature between 473 and 673 K for 1 h under an Ar gas flow. b) XRD profile of β‐SnMoO_4_ prepared at 498 K (black cross) and the calculated profiles obtained by Rietveld analysis (red solid line). Solid blue line corresponds to the difference between the observed and the calculated intensities. Vertical ticks indicate the positions of the Bragg reflections. c) Refined structural parameters for β‐SnMoO_4_: space group type of *P*2_1_3 (No. 198), lattice constants of *a* = 7.263(7) Å, *R*_wp_ = 3.21%, *R*_p_ = 2.47%, and *S*_fit_ = 3.57 (see Figure S1 in the Supporting Information for more details).

In the present study, we synthesized β‐SnMoO_4_ after many trials using various starting materials and synthesis conditions. **Figure** **3**a shows the XRD profiles of the samples obtained by reacting SnCl_2_ and K_2_MoO_4_ powders at a constant temperature between 473 and 673 K for 1 h under an Ar gas flow. After cooling to room temperature, the product was washed with distilled water and dried at 323 K. For the sample treated at 473 K, both SnCl_2_ and KCl peaks appear despite washing. In contrast, the sample treated at 488 K results in sharp peaks, which are attributed to β‐SnMoO_4_. Moreover, an exothermic reaction is detected between 473 and 488 K by differential thermal analysis, implying that a chemical reaction forms β‐SnMoO_4_. For samples prepared above 573 K, the secondary phase is predominant. This secondary phase is assigned to the rutile‐like SnO_2_‐MoO_2_ solid solution with tetragonal lattice constants of *a* = *b* = 4.797 and *c* = 2.995 Å. Increasing the temperature to 773 K results in decomposition to SnO_2_ and MoO_2_, suggesting that the rutile‐like SnO_2_‐MoO_2_ solid solution is a metastable phase.

The lattice constant and internal coordinates of β‐SnMoO_4_ were analyzed by the Rietveld method using the XRD profile of the sample prepared at 498 K (Figure 3b,c and Figure S3 (Supporting Information)). The cell parameter for β‐SnMoO_4_ (7.263 Å) is 4% smaller than the DFT lattice constant (7.543 Å). Both the cell parameter and the internal coordinate of each atom in β‐SnMoO_4_ are similar to those in β‐SnWO_4_. The powder is bright brown (Figure 3b, inset), implying that β‐SnMoO_4_ has a band gap within the visible region. The Sn/Mo ratio obtained by the energy‐dispersive X‐ray spectrum analyzer on a scanning electron microscope (SEM‐EDX) analysis is 0.96 ± 0.08.

**Figure 4:**
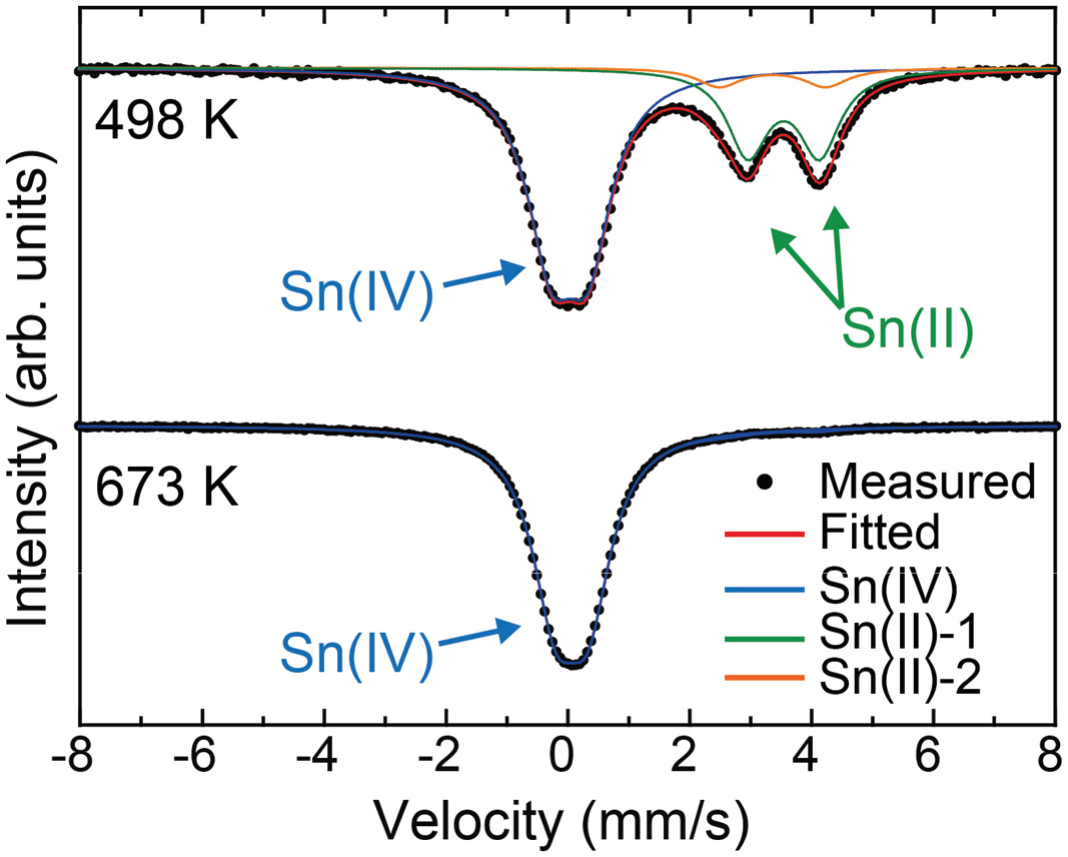
^119^Sn Mössbauer spectra of the samples prepared at 498 and 673 K. Black dots indicate experimental spectra. Solid lines represent the fitted curves.

Mössbauer experiments were performed at room temperature to examine the chemical state of Sn. Unlike the sample heated at 673 K, which is entirely in the Sn(IV) state, the sample heated at 498 K has a significant fraction of Sn in the Sn(II) state as shown in **Figure** **4**. The experimental spectrum of the sample heated at 498 K can be decomposed into three components: (1) Sn(II)‐1 (isomer shift *δ* = 3.54 mm s^−1^, relative to CaSnO_3_, quadrupole splitting *Δ* = 1.18 mm s^−1^), (2) Sn(II)‐2 (*δ* = 3.36 mm s^−1^, *Δ* = 1.75 mm s^−1^), and (3) Sn(IV) (*δ* = 0.03 mm s^−1^, *Δ* = 0.61 mm s^−1^). Considering the fact that β‐SnMoO_4_ with a Sn(II) coordination environment is predominant in the XRD of the sample heated at 498 K, spectrum (1) is assigned to crystalline β‐SnMoO_4_ with a single Sn site. The large *Δ* can be well explained by the trigonal pyramidal coordination environment of the Sn(II) atoms with a large electric field gradient at the Sn nucleus. The origin of spectrum (2) (areal intensity: 7%) is unclear. Spectrum (3) may be assigned to the nanocrystalline SnO_2_‐MoO_2_ solid solution with Sn(IV). There is evidence of a nanocrystalline SnO_2_‐MoO_2_ solid solution even in the XRD profile as described in Figure S1 (Supporting Information). Although the areal intensity of spectrum (1) is 28% of the total absorption, the content of β‐SnMoO_4_ should be larger because of the stronger temperature dependence of the recoil‐free fraction of Sn(II) oxides than that of Sn(IV) oxides.^30^ On the other hand, the experimental spectrum of the sample heated at 673 K can be assigned entirely to Sn(IV) oxides, which is consistent with the XRD peaks in Figure 2a that are identified as a solid solution of SnO_2_‐MoO_2._

### 2.4 Photocatalytic Activity

**Figure 5:**
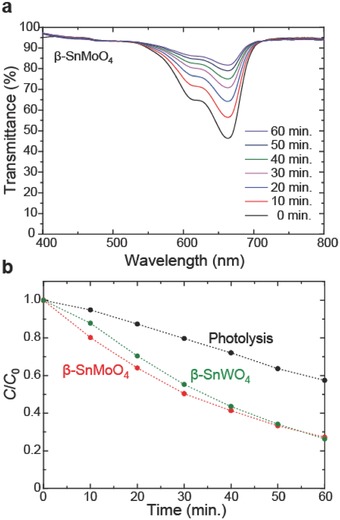
Photocatalytic activity of β‐SnMoO_4_. a) Transmittance spectra of a MB solution with β‐SnMoO_4_ powder measured at room temperature. Peaks near 664 nm correspond to the absorption of MB. b) Irradiation time dependence of the relative concentration of MB solutions with and without β‐SnMoO_4_ and β‐SnWO_4_ powders.

The photocatalytic activity of β‐SnMoO_4_ powder was evaluated via the degradation of a methylene blue (MB) solution under simulated daylight irradiation. For comparison, β‐SnWO_4_ powder synthesized by a similar chemical route was also used for the photocatalytic activity test. **Figure** **5**a shows the transmittance spectra of the MB solution with the β‐SnMoO_4_ powder measured at room temperature. The peaks near 664 nm correspond to the absorption of MB. The intensities of these peaks monotonically decrease upon daylight irradiation.

Figure 5b plots the relative concentration of the MB solution as a function of the irradiation time along with the results by MB photolysis measured under the same irradiation conditions without supplying an oxide powder. The photocatalytic activity of β‐SnMoO_4_ is clear. Note that the relative concentration of the MB solution decreases only by 4% without irradiation as shown in Figure S4 (Supporting Information). It also should be noted that the XRD profiles and the particle size of β‐SnMoO_4_ and β‐SnWO_4_ powders are similar to each other as shown in Figures S5 and S6 (Supporting Information). These results imply that the photocatalytic activity of β‐SnMoO_4_ powder is at least as good as that of β‐SnWO_4_, which exhibits a comparable photocatalytic activity with BiVO_4_.^22^ The photocatalytic activity of BiVO_4_ under the same experimental setup is comparatively shown in Figure S7 (Supporting Information).

## 3 Conclusions

Through a rational search of 3483 candidate Sn(II)‐based oxides, synthesis experiments, and detailed characterization, we discovered a novel oxide photocatalyst, β‐SnMoO_4_. DFT calculations were performed for the whole range of SnO‐*M*O*_q_*_/2_ [*M*: Ti, Zr, and Hf (*q* = 4); V, Nb, and Ta (*q* = 5); Cr, Mo, and W (*q* = 6)] pseudobinary systems with diverse compositions and structures. Initially, the thermodynamic stability was screened, revealing that 28 compounds are located either on the convex hull or within 5 meV per atom above the convex hull (Table S1, Supporting Information). DFPT calculations of these 28 compounds indicate that only one has imaginary phonon modes at the Γ point, suggesting that the other 27 possess dynamic stability for the Γ point phonon mode. Then, the electronic structures of these thermodynamically stable ternary compounds were screened using the band gap and the band‐edge positions as descriptors. Seven potential photocatalyst candidates have favorable band‐edge positions.

Next we examined β‐SnMoO_4_, an unknown compound in detail. First, we developed a successful low temperature synthesis, which was confirmed by X‐ray powder diffraction and Mössbauer spectroscopy. The activity of this material for the photocatalytic decomposition of the MB solution under daylight is as good as other well‐studied oxide photocatalysts of *n*s^2^ cations, such as β‐SnWO_4_ and BiVO_4_. In addition to revealing a novel photocatalyst, this study demonstrates that a combination of high throughput screening using a large DFT database and subsequent focused synthesis experiments is a powerful tool for accelerated discovery of novel oxide photocatalysts.

## 4 Experimental Section

*DFT Calculations*: All DFT calculations were performed using the projector augmented‐wave (PAW) method as implemented in the Vienna Ab initio simulation package (VASP) code.^31^, ^32^ The Perdew–Burke–Ernzerhof generalized gradient approximation (PBE‐GGA)^33^ was used for the screening. Table S1 (Supporting Information) summarized the calculated band gaps along with the values obtained using other approximations, including the PBEsol GGA,^34^ Tao–Perdew–Staroverov–Scuseria meta‐GGA,^35^ and Heyd–Scuseria–Ernzerhof 06 hybrid functional^36^, ^37^, ^38^ and experimental values. The PAW data sets with radial cutoffs of 1.6, 1.5, 1.5, and 0.8 Å for Sn, Mo, W, and O, respectively, were used with a plane‐wave cutoff energy of 550 eV. The Sn 5s, 5p; Mo 4p, 5s, 4d; W 6s, 5d; and O 2s, 2p states were described as valence electrons.

The crystal structures were taken from the ICSD.^23^ The lattice parameters and internal coordinates were fully relaxed until the residual stresses and forces converged to less than 0.1 GPa and 0.01 eV Å^−1^. *k*‐point sampling mesh was determined by checking the convergence of the total energy for each calculation. The optical absorption spectra were acquired via calculations of the dielectric functions within the independent particle approximation. The electron DOS and dielectric functions for β‐Sn*M*O_4_ (*M* = Mo and W) were calculated using a 6 × 6 × 6 *k*‐point mesh. The DOS was calculated using the tetrahedron method with corrections of Blöchl et al.^39^ Bader charge analyses were carried out using the Bader code^40^, ^41^, ^42^ with the charge density obtained from DFT calculations. The ionization potential (IP) and electron affinity (EA), which correspond to the VBM and the CBM against the vacuum level, respectively, were calculated using the bulk‐based definition according to ref. 43. Slab‐vacuum models with slab and vacuum thicknesses of ≥≈20 Å were obtained using the algorithm in ref. 44. Low index nonpolar surfaces were systematically investigated, and the IP and EA values for the lowest surface energy were adopted.

*Materials Synthesis*: Polycrystalline β‐SnMoO_4_ powders were prepared by a low temperature synthesis using reagent‐grade SnCl_2_ and K_2_MoO_4_ powders (Kojundo Chemical Lab. (Japan)), which were well dried at 373 K in a dry Ar gas. Stoichiometric mixtures of the starting materials were ground in an Ar atmosphere. After pelletization, the specimens were placed in an alumina crucible and heated between 473 and 673 K for 1 h under Ar gas flow. After cooling to room temperature, the product was washed with distilled water and dried at 323 K. β‐SnWO_4_ was prepared in a similar manner with a heating temperature of 483 K using K_2_WO_4_ as the raw material.

*Characterization*: The crystal phases and structures were determined using an X‐ray diffractometer. The lattice parameters and atomic coordinates were obtained using RIETAN FP Rietveld refinement software. The composition was measured using an SEM‐EDX. Transmission^119^Sn Mössbauer measurements with a Ca^119^SnO_3_ gamma‐ray source were conducted in the constant acceleration mode at room temperature. The MB solution was photocatalytically degraded under simulated daylight using 300 W xenon lamps with a cutoff filter (*λ* > 350 nm). The irradiance and irradiation angle to the water surface were set to be 400 W m^−2^ (0.4 “SUN”) and 30°. The powder (1.9 mg of β‐SnMoO_4_ and 2.0 mg of β‐SnWO_4_) was fixed at the bottom of the reaction cell (20 × 20 × 10 mm) with 2 mL of the MB solution (2 × 10^−2^ mol m^−3^) by double‐faced tape. The transmittance spectra of the sample solutions were measured every 10 min using a UV–vis spectrophotometer.

## Supporting information

As a service to our authors and readers, this journal provides supporting information supplied by the authors. Such materials are peer reviewed and may be re‐organized for online delivery, but are not copy‐edited or typeset. Technical support issues arising from supporting information (other than missing files) should be addressed to the authors.

Supplementary
