# Supplementary material for: Discovery of a Novel Sn(II)‐Based Oxide β‐SnMoO4 for Daylight‐Driven Photocatalysis
Source: Adv Sci (Weinh). 2016 Sep 8;4(1):1600246. doi: 10.1002/advs.201600246 (PMC5238750; doi:10.1002/advs.201600246)
Supplement: Supplementary file 1 — Supplementary [file ADVS-4-0-s001.pdf]

Copyright WILEY-VCH Verlag GmbH & Co. KGaA, 69469 Weinheim, Germany, 2016.

ADVANCED SCIENCE
Open Access

Supporting Information

for *Adv. Sci.,* DOI: 10.1002/advs.201600246

Discovery of a Novel Sn(II)-Based Oxide β-$SnMoO_4$ for Daylight-Driven Photocatalysis

Hiroyuki Hayashi, Shota Katayama, Takahiro Komura, Yoyo Hinuma, Tomoyasu Yokoyama, Ko Mibu, Fumiyasu Oba, and Isao Tanaka**

Supporting information

Discovery of a novel Sn(II)-based oxide β-$SnMoO_4$ for daylight-driven photocatalysis

Hiroyuki Hayashi, Shota Katayama, Takahiro Komura, Yoyo Hinuma,*
*Tomoyasu Yokoyama, Ko Mibu, Fumiyasu Oba, and Isao Tanaka**

Dr. H. Hayashi, Dr. S. Katayama, T. Komura, Dr. Y. Hinuma, T. Yokoyama, Prof. F. Oba, Prof. I. Tanaka
Department of Materials Science and Engineering, Kyoto University, Sakyo, Kyoto 606-8501, Japan
Correspondence and requests for materials should be addressed to
H.H. (email: hayashi.hiroyuki.5w@gmail.com) and I.T. (email: tanaka@cms.mtl.kyoto-u.ac.jp).

Prof. K. Mibu
Graduate School of Engineering, Nagoya Institute of Technology, Showa-ku, Nagoya, Aichi 466-8555, Japan

Prof. F. Oba
Materials and Structures Laboratory, Tokyo Institute of Technology, Yokohama 226-8503, Japan

Dr. H. Hayashi, Dr. Y. Hinuma, Prof. F. Oba, Prof. I. Tanaka
Center for Materials Research by Information Integration, National Institute for Materials Science, Tsukuba 305-0047, Japan

Prof. I. Tanaka
Nanostructures Research Laboratory, Japan Fine Ceramics Center, Nagoya 456-8587, Japan

Supplementary Note 1: Crystal structures and band gaps of thermodynamically stable compounds

Table S1 summarizes the crystal structures and band gaps of thermodynamically stable compounds in SnO-$MO_{q/2}$ [M: Ti, Zr, and Hf (q=4); V, Nb, and Ta (q=5); Cr, Mo, and W (q=6)] pseudo-binary systems. Note that the ICSD number of an isostructural compound is shown only when the ICSD prototype is unavailable. These ICSD prototype structures are used as the initial structures in the DFT calculations. The space group types of the structures after structural optimization are determined using phonopy code [1].

In addition, Table S1 shows the theoretical band gaps obtained using the PBE [2] and PBEsol [3] GGA functionals, the TPSS meta-GGA functional [4], and the HSE06 hybrid functional [5-7] along with available experimental values [8-13]. Although the HSE06 hybrid functional has been reported to improve band gaps over the GGA for many semiconductors and insulators with relatively simple electronic structures [7,14], it overestimates the band gaps of $SnWO_4$, especially for the β-phase ($P2_13$). PBE-GGA yields values closer to the experiments. Given the similarity in the electronic structures between β-$SnMoO_4$ and β-$SnWO_4$, which are the main targets of the present study, Figs. 1 and 2 show the band structures and band positions obtained using PBE-GGA.

Table S1. Thermodynamically stable compounds in SnO-$MO_{q/2}$ (M: Ti, Zr, and Hf (q=4); V, Nb, and Ta (q=5); Cr, Mo, and W (q=6)) pseudo-binary systems. Unknowns are 22 compounds without ICSD numbers. Theoretical band gaps obtained using the PBE [2] and PBEsol [3] GGA functionals, the TPSS meta-GGA functional [4], and the HSE06 hybrid functional [5-7] are shown along with the experimental values [8-13].

.

Chemical formula	ICSD number	ICSD prototype structure	Space group type	Energy above convex hull, PBE-GGA (meV/atom)	E_g (eV)				
					PBE	PBEsol	TPSS	HSE06	Exp.
Sn_2TiO_4		Pb_3O_4(LT)	*Pbam*	1	0.9	0.8	0.9	1.7	
Sn_2TiO_4	163230	Pb_3O_4	$P4_2/mbc$	1	0.9	0.8	0.9	1.7	
$SnTiO_3$		$FePS_3$	$C2/m$	0	1.4	0.9	1.6	2.2	
$SnTiO_3$		$Hg_2P_2Se_6$	$C2/c$	0	1.4	0.9	1.6	2.2	
$SnTiO_3$		$FeTiO_3$	$R\bar{3}$	0	1.3	0.9	1.3	2.1	
$SnTiO_3$		$AlSiTe_3$	$P\bar{3}1m$	0	1.7	1.5	1.8	2.7	
$Sn_2V_2O_7$		Ti_4O_7	$P\bar{1}$	2	1.8	1.5	1.7	2.6	
$Sn_2V_2O_7$		170847	$P2_1/c$	0	1.8	1.5	1.7	2.7	
$Sn_2V_2O_7$		$Y_2Si_2O_7$	$P2_1/c$	3	1.3	1.0	1.2	2.3	
$Sn_5V_2O_{10}$		418458	$P\bar{1}$	0	1.8	1.4	1.6	2.6	
SnV_2O_6		CuV_2O_6	$C2$	0	1.4	1.3	1.3	2.3	
SnV_2O_6		$Pb(PO_3)_2$	$P2_1/c$	3	1.6	1.4	1.5	2.5	
SnV_4O_{11}		MgP_4O_{11}	$P2_1/c$	0	0.0	0.0	0.0	0.7	
$SnNb_2O_6$		54078	*Cc*	0	1.7	1.5	1.6	2.4	
$SnNb_2O_6$	202827	Nb_2SnO_6	$C2/c$	0	1.7	1.5	1.6	2.4	2.3[8], 2.6[9]
$SnTa_2O_6$	54078	54078	*Cc*	0	2.2	2.0	2.0	3.0	
$SnTa_2O_6$		Nb_2SnO_6	$C2/c$	0	2.2	2.0	2.0	3.0	3.0[10]
$SnTa_4O_{11}$		$CaTa_4O_{11}$	$P6_322$	2	2.4	2.3	2.4	3.3	
Sn_2CrO_5		35101	$P\bar{4}2_1c$	0	1.2	1.0	1.1	1.9	
$SnCr_2O_7$*		CdS_2O_7	$P2_1/c$	0	0.0	0.0	0.0	1.5	
$SnCrO_4$		$BaZnCl_4$	*Pbcn*	0	0.0	0.0	0.0	1.3	
Sn_2MoO_5		35101	$P\bar{4}2_1c$	0	2.3	2.1	2.3	3.1	
$SnMo_2O_7$		$CsSb_2F_7$	$C2/c$	0	0.0	0.0	0.0	0.0	
$SnMoO_4$		$SnWO_4$	$P2_13$	0	3.0	2.7	2.9	3.9	
Sn_2WO_5	249546	249546	$P2_1/c$	0	2.0	1.8	2.0	2.9	
$SnWO_4$		Sb_3TaO_8	$Pna2_1$	2	1.3	0.8	0.8	2.1	
$SnWO_4$	2147	$SnWO_4$($oP24$)	*Pnna*	2	1.3	0.8	0.8	2.1	1.6[11], 1.9[12]
$SnWO_4$	2840	$SnWO_4$	$P2_13$	0	3.7	3.5	3.7	4.8	2.7[11], 2.6-3.4[13]

*) Dynamically unstable for the Γ-point phonon mode.

Table S2. Experimentally reported compounds in pseudo-binary SnO-$MO_{q/2}$ (M: Ti, Zr, and Hf (q=4); V, Nb, and Ta (q=5); Cr, Mo, and W (q=6)) systems. Eight compounds are registered in the ICSD. Four compounds are taken from Ref. [10,15-17].

Compound	ICSD number	ICSD prototype structure	Space group type
Sn_2TiO_4	163230	Pb_3O_4	$P4_2/mbc$
$SnTiO_3$	[15]		$R\bar{3}$
$SnNb_2O_6$	202827	Nb_2SnO_6	$C2/c$
$Sn_2Nb_2O_7$	[16]		$Fd\bar{3}m$
$SnTa_2O_6$	54078	54078	Cc
	[10]		$C2/c$
$Sn_2Ta_2O_7$	27119	Pyrochore-NaCa(Nb_2O_6)F	$Fd\bar{3}m$
$SnTa_4O_{11}$	[17]		unknown
Sn_3WO_6	249534		$C2/c$
Sn_2WO_5	249546		$P2_1/c$
α-$SnWO_4$	2147	$SnWO_4$($oP24$)	$Pnna$
β-$SnWO_4$	2840	$SnWO_4$	$P2_13$

Table S3. Calculated ionization potential (IP) and electron affinity (EA) for the seven compounds remaining after the band gap screening. Crystallographic conventional cells are used in the slab models. VBM and CBM with respect to the vacuum level shown in Fig. 1 correspond to the negatives of IP and EA, respectively.

Chemical formula	Space group type	Plane	Surface energy (meV/Å^2)	Ionization potential (eV)	Electron affinity (eV)
$SnTa_2O_6$	Cc	(110)	39	-6.2	-4.0
$SnTa_2O_6$	$C2/c$	(110)	39	-6.2	-4.0
$SnTa_4O_{11}$	$P6_322$	$(10\bar{1}0)$	62	-6.2	-3.8
Sn_2MoO_5	$P\bar{4}2_1c$	(100)	11	-5.7	-3.5
$SnMoO_4$	$P2_13$	(100)	10	-7.0	-4.0
Sn_2WO_5	$P2_1/c$	(010)	3	-5.9	-3.9
$SnWO_4$	$P2_13$	(100)	15	-7.1	-3.3

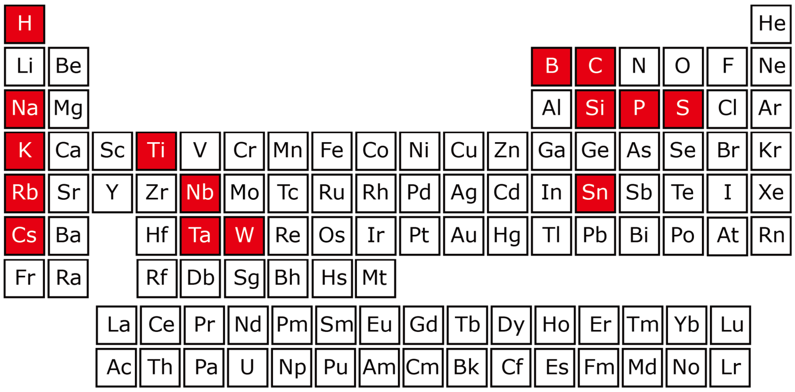

Figure S1. Elements in red are registered in the ICSD to form ternary oxides with Sn(II).

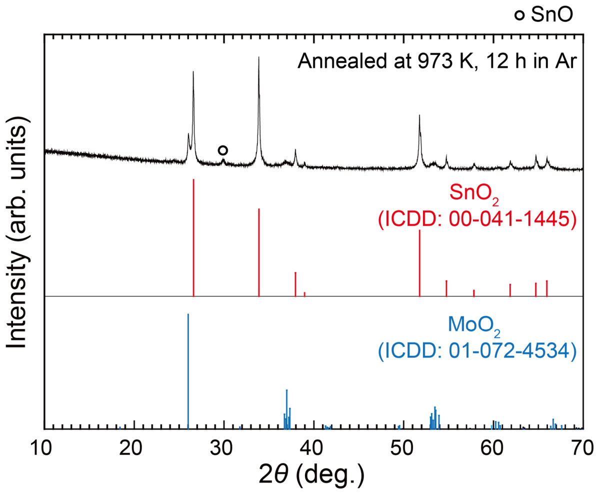

Figure S2. XRD profiles for the sample prepared by a solid state reaction route comparing with the XRD peak positions of SnO_2 (ICDD: 00-041-1445) and MoO_2 (ICDD: 01-072-4534). The mixed powder of SnO and MoO_3 was annealed at 973 K in an Ar atmosphere for 12 hours.

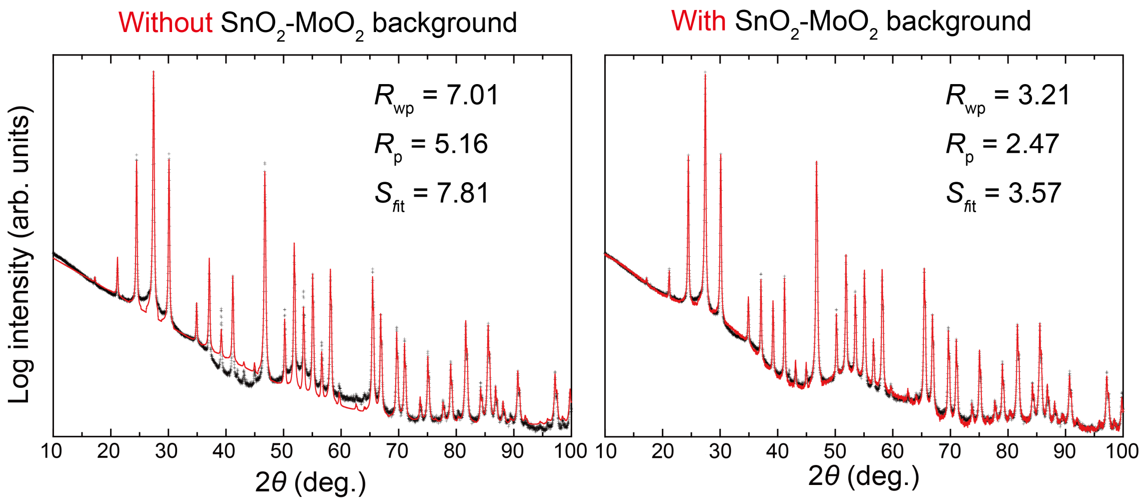

Figure S3. Rietveld analysis of the XRD profile for the sample heated at 498 K with a log scale. (Left) Result with β-$SnMoO_4$ only. (Right) Result with β-$SnMoO_4$ and a nanocrystalline SnO_2-MoO_2 solid solution. Including the contribution of the nanocrystalline SnO_2-MoO_2 solid solution significantly reduces the fitting error. Presence of the nanocrystalline SnO_2-MoO_2 solid solution is consistent with Mössbauer experimental results.

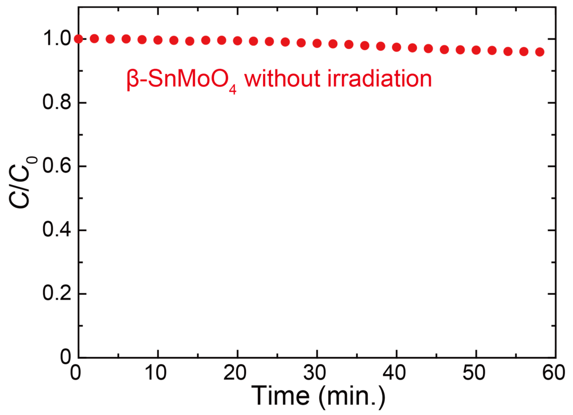

Figure S4. The relative concentration of MB solutions with the β-$SnMoO_4$ powder against elapsed time without irradiation. It shows an approximately 4% decline of MB solutions over 1 hour.

Comparative experiments with $BiVO_4$ powder and TiO_2 (Degussa P25)

Polycrystalline monoclinic $BiVO_4$ powders were prepared by a solid state reaction route using reagent-grade Bi_2O_3 and V_2O_5 powders (Kojundo Chemical Lab. (Japan)). Stoichiometric mixtures of the starting materials were well ground at room temperature in air. After pelletization, the specimens were placed in an alumina crucible and annealed at 1173 K for 16 hours in air. After the annealing, the product was furnace cooled down to room temperature. The product was ground by a planetary mill to measure the photocatalytic activity.

As shown in Figure S7, the photocatalytic activity of β-$SnMoO_4$ is higher than TiO_2 (P25) and $BiVO_4$. It should be noted, however, further works are necessary to quantify the activity in detail, since the comparison was made with powders synthesized by different methods.

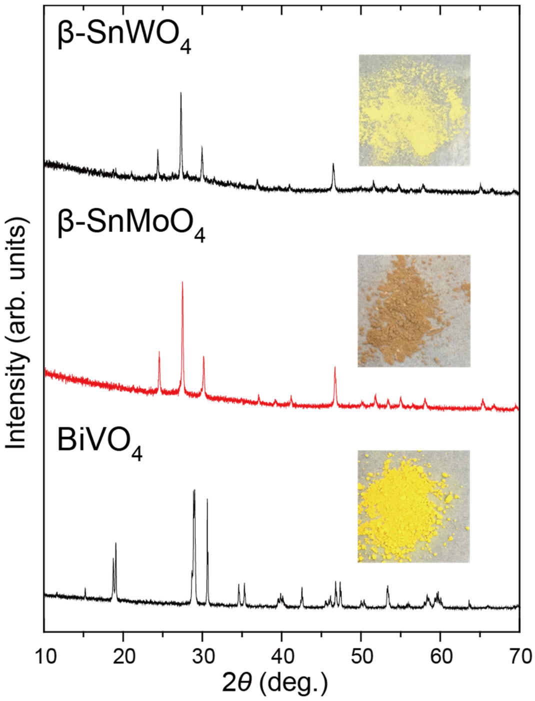

Figure S5. XRD profiles for the β-$SnWO_4$, β-$SnMoO_4$ and $BiVO_4$ powders. (Upper) β-$SnWO_4$ prepared at 483 K, (Middle) β-$SnMoO_4$ prepared at 498 K and (Lower) $BiVO_4$ prepared at 1173 K. The insets are the pictures of β-$SnWO_4$, β-$SnMoO_4$ and $BiVO_4$ powders, respectively.

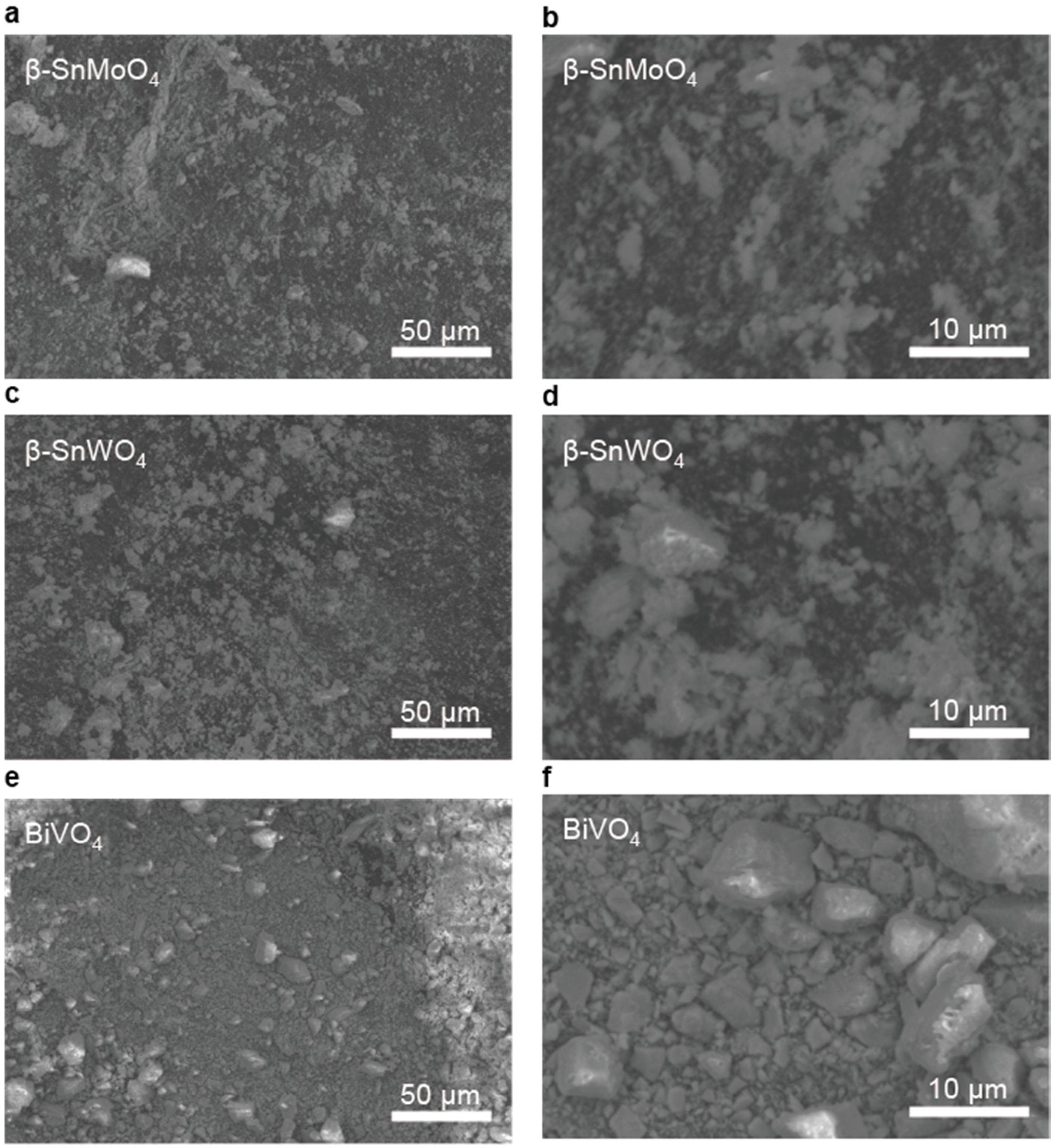

Figure S6. SEM images for the β-$SnWO_4$, β-$SnMoO_4$ and $BiVO_4$ powders. (a) β-$SnMoO_4$ prepared at 498 K, (c) β-$SnWO_4$ prepared at 483 K, and (e) $BiVO_4$ prepared at 1173 K. (b), (d) and (f) are the magnified view of (a), (c), and (e), respectively.

Figure S7. Photocatalytic activity and rate constants of photolysis, β-$SnMoO_4$, β-$SnWO_4$, TiO_2 (Degussa P25), and $BiVO_4$. (a) Irradiation time dependence of the relative concentration of MB solutions with and without $BiVO_4$, TiO_2 (P25), β-$SnMoO_4$ and β-$SnWO_4$ powders. (b) The photocatalytic rate constants of each sample. The kinetics of the degradation reaction were fitted using the equation $\ln(C/C_0) = -kt$, where k is the apparent rate constant and t is the irradiation time.

a

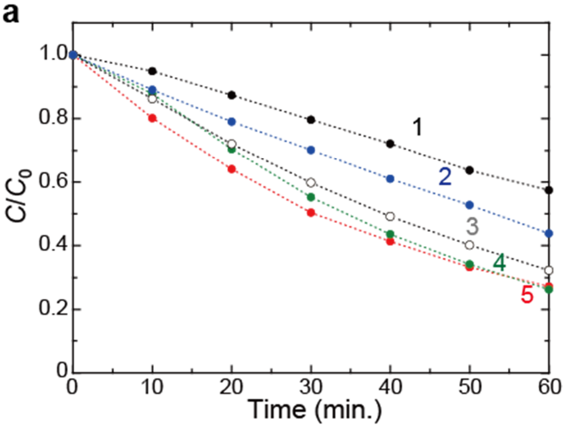

b

Number	Sample	Rate constant k (min^{-1})
1	Photolysis	0.009
2	$BiVO_4$	0.013
3	TiO_2 (P25)	0.018
4	β-$SnWO_4$	0.021
5	β-$SnMoO_4$	0.022

References

1 Togo, A. & Tanaka, I. First principles phonon calculations in materials science. *Scr. Mater.*, **108**, 1-5 (2015).

2 Perdew, J. P., Burke, K., & Ernzerhof, M. Generalized Gradient Approximation Made Simple. *Phys. Rev. Lett.* **77**, 3865-3868, (1996).

3 Perdew, J. P. *et al.* Restoring the Density-Gradient Expansion for Exchange in Solids and Surfaces. *Phys. Rev. Lett.* **100**, 136406, (2008).

4 Sun, J. *et al.* Self-consistent meta-generalized gradient approximation within the projector-augmented-wave method. *Phys. Rev. B* **84**, 035117, (2011).

5 Heyd, J., Scuseria, G. E. & Ernzerhof, M. Hybrid functionals based on a screened Coulomb potential. *J. Chem. Phys.* **118**, 8207, (2003).

6 Heyd, J., Scuseria, G. E. & Ernzerhof, M. Erratum: "Hybrid functionals based on a screened Coulomb potential" [J. Chem.Phys.118, 8207 (2003)]. *J. Chem. Phys.* **124**, 219906, (2006).

7 Krukau, A. V., Vydrov, O. A., Izmaylov, A. F. & Scuseria, G. E. Influence of the exchange screening parameter on the performance of screened hybrid functionals. *J. Chem. Phys.* **125**, 224106, (2006).

8 Hosogi, Y., Tanabe, K., Kato, H., Kobayashi, H. & Kudo, A. Energy Structure and Photocatalytic Activity of Niobates and Tantalates Containing Sn(II) with a $5s^2$ Electron Configuration. *Chem. Lett.* **33**, 28-29, (2004).

9 Liang, S., Zhu, S., Chen, Y., Wu, W., Wang, X. & Wu, L. Rapid template-free synthesis and photocatalytic performance of visible light-activated $SnNb_2O_6$ nanosheets. *J. Mater. Chem.* **22**, 2670–2678, (2012).

10 Hosogi, Y., Shimodaira, Y., Kato, H., Kobayashi, H. & Kudo, A. Role of Sn^{2+} in the Band Structure of SnM_2O_6 and $Sn_2M_2O_7$ (M = Nb and Ta) and Their Photocatalytic Properties. *Chem. Mater.* **20**, 1299-1307, (2008).

11 Cho, I., Kwak, C. H., Kim, D. W., Lee, S. & Hong, K. S. Photophysical, Photoelectrochemical, and Photocatalytic Properties of Novel $SnWO_4$ Oxide Semiconductors with Narrow Band Gaps. *J. Phys. Chem. C* **113**, 10647-10653, (2009).

12 Su, Y., Hou, L., Du, C., Peng, L., Guan, K. & Wang X. Rapid synthesis of Zn^{2+} doped $SnWO_4$ nanowires with the aim of exploring doping effects on highly enhanced visible photocatalytic activities. *RSC Adv.* **2**, 6266-6273, (2012).

13 Stoltzfus, M. W., Woodward, P. M., Seshadri, R., Klepeis, J. & Bursten, B. Structure and Bonding in $SnWO_4$, $PbWO_4$, and $BiVO_4$: Lone Pairs vs Inert Pairs. *Inorg. Chem.* **46**, 3839-3850, (2007).

14 Paier, J., Marsman, M., Hummer, K., Kresse, G., Gerber, I. C. & Angyan, J. G. Screened hybrid density functionals applied to solids. *J. Chem. Phys*. **124**, 154709 (2006).

15 Fix, T., Sahonta, S. L., Garcia, V., MacManus-Driscoll, J. L. & Blamire, M. G. Structural and Dielectric Properties of $SnTiO_3$, a Putative Ferroelectric. *Cryst. Growth Des.* **11**, 1422-1426, (2011).

16 Cruz, L. P., Savariault, J. M., Rocha, J., Jumas, J. C. & Pedrosa de Jesus, J. D. Synthesis and Characterization of Tin Niobates. *J. Solid State Chem.* **156**, 349-354, (2001).

17 Bodiot, D. Comparison of Elements Niobium and Tantalium from Compounds Yields by Solid State Reactions between Their Hemipentoxides and Oxides SnO, CeO_2, UO_2, or M_2O_3 (M= Lanthanoid). *Rev. Chim. Miner.* **5**, 569, (1968).
